# Congenital Tufting Enteropathy-Associated Mutant of Epithelial Cell Adhesion Molecule Activates the Unfolded Protein Response in a Murine Model of the Disease

**DOI:** 10.3390/cells9040946

**Published:** 2020-04-11

**Authors:** Barun Das, Kevin Okamoto, John Rabalais, Ronald R. Marchelletta, Kim E. Barrett, Soumita Das, Maho Niwa, Mamata Sivagnanam

**Affiliations:** 1 Department of Pediatrics, University of California, San Diego, CA 92093, USA; bdas@health.ucsd.edu (B.D.); kokamoto@health.ucsd.edu (K.O.); jrabalai@gcloud.ucsd.edu (J.R.); 2 Department of Medicine, University of California, San Diego, CA 92093, USA; ronmarchelletta@hotmail.com (R.R.M.); kbarrett@health.ucsd.edu (K.E.B.); 3 Department of Pathology, University of California, San Diego, CA 92093, USA; sodas@health.ucsd.edu; 4 Division of Biological Sciences, University of California, San Diego, CA 92093, USA; mniwarosen@ucsd.edu; 5 Rady Children’s Hospital, San Diego, CA 92123, USA

**Keywords:** congenital tufting enteropathy, mutant EpCAM, ER stress, unfolded protein response

## Abstract

Congenital tufting enteropathy (CTE) is a rare chronic diarrheal disease of infancy caused by mutations in epithelial cell adhesion molecule (EpCAM). Previously, a murine CTE model showed mis-localization of EpCAM away from the basolateral cell surface in the intestine. Here we demonstrate that mutant EpCAM accumulated in the endoplasmic reticulum (ER) where it co-localized with ER chaperone, GRP78/BiP, revealing potential involvement of ER stress-induced unfolded protein response (UPR) pathway in CTE. To investigate the significance of ER-localized mutant EpCAM in CTE, activation of the three UPR signaling branches initiated by the ER transmembrane protein components IRE1, PERK, and ATF6 was tested. A significant reduction in BLOS1 and SCARA3 mRNA levels in EpCAM mutant intestinal cells demonstrated that regulated IRE1-dependent decay (RIDD) was activated. However, IRE1 dependent *XBP1* mRNA splicing was not induced. Furthermore, an increase in nuclear-localized ATF6 in mutant intestinal tissues revealed activation of the ATF6-signaling arm. Finally, an increase in both the phosphorylated form of the translation initiation factor, eIF2α, and ATF4 expression in the mutant intestine provided support for activation of the PERK-mediated pathway. Our results are consistent with a significant role for UPR in gastrointestinal homeostasis and provide a working model for CTE pathophysiology.

## 1. Introduction

Congenital tufting enteropathy (CTE) belongs to a family of congenital diarrhea and enteropathies that cause persistent and severe diarrhea in infants and often lead to life-threatening intestinal failure. It is characterized by partial villous atrophy, crypt hyperplasia, and focal “tufts” of enterocytes of the intestinal epithelium [1,2]. Most patients with CTE depend on parenteral nutrition and no direct treatments are currently available for the management of this disease. Thus, there is an urgent need to understand the pathophysiology of CTE to allow for the development of CTE therapeutics.

Mutations in EPCAM (Epithelial Cell Adhesion Molecule; also known as CD326, GA773-2, TACSTD1, KSA, and TROP-1) were identified as causative in CTE [3]. EpCAM is a cell surface glycoprotein that is highly expressed on the basolateral membrane of most epithelia, but with the highest expression in the gut [4,5]. Amongst the 42 distinct reported mutations of EPCAM that cause CTE, most result in the expression of mutant EpCAM protein [6,7]. Patients with a frameshift mutation or truncation of the gene are reported to have a severe disease phenotype [6]. Based on the X-ray crystal structure of wild-type full-length EpCAM [8], mutations resulting in the deletion of exon 4 of EPCAM truncate the core region of the protein’s C terminal domain [6].

Despite EpCAM’s tight link to CTE, the detailed molecular and functional mechanisms underlying disease phenotypes remain elusive. Studies using HEK 293 cells expressing several forms of mutant EpCAM that cause CTE demonstrate that these mutant forms are not routed to their normal plasma membrane location, but rather are sequestered in the endoplasmic reticulum (ER) [7]. While these results in tissue culture are suggestive of the aberrantly-localized EpCAM found in CTE patients’ biopsy samples and a mutant EpCAM mouse model, the precise localization of mutant EpCAM in CTE patient tissue has not yet been identified [9]. Proteins destined to be localized to the cell surface, such as EpCAM, enter the ER lumen as linear nascent polypeptides and are then targeted for proper folding, N-glycosylation, di-sulfide linkage formation, and other post-translational modifications [10]. The ER is equipped with a quality control mechanism whereby only properly folded and modified proteins can exit and ultimately reach their final destinations [11]. The accumulation of misfolded proteins in the ER is harmful to cells and thus, the ER has evolved mechanisms designed to detect misfolded proteins and either refold them or target them for degradation [12]. Therefore, the overall function of the ER can be adjusted to increase ER capacity via a mechanism called the unfolded protein response (UPR). Apart from well-established links to several neurodegenerative and cancer pathophysiologies [13,14,15], ER stress and UPR have been shown to have direct effects on intestinal physiology in several studies, including cystic fibrosis [16] and inflammatory bowel disease (IBD) pathogenesis [17,18]. The UPR is initiated by three highly-conserved ER transmembrane components, Inositol-requiring enzyme 1 (IRE1), protein kinase RNA-like endoplasmic reticulum kinase (PERK), and activating transcription factor 6 (ATF6).

As all three arms of the UPR are transduced in parallel, they can systematically and independently act to alleviate the stress response. Splicing of X-box binding protein 1 (XBP1) mRNA by activated IRE1 generates an active form of the transcription factor, spliced XBP1 (sXBP1), which in turn induces transcription of genes that are critical to resolve ER stress [19]. Additionally, IRE1 RNase selectively cleaves ER bound mRNAs such as scavenger receptor class A, member 3 (SCARA3) and biogenesis of lysosome-related organelles complex 1 subunit 1 (BLOS1), resulting in their degradation to alleviate the protein load by a process called regulated IRE1 dependent degradation (RIDD) [20]. Upon recognition of accumulated misfolded proteins, another ER stress transducer, PERK is activated and phosphorylates eukaryotic translation initiation factor 2 alpha (eIF2α) in order to attenuate global protein translation, thereby decreasing ER protein burden and restoring ER homeostasis [21]. While attenuating global translation, phosphorylated eIF2α facilitates the translation of its downstream transcription factor, activating transcription factor 4 (ATF4), via small open reading frames localized within its 5′ UTR. ATF4, in turn, activates the transcription of genes involved in re-establishment of functional UPR [22]. Upon induction of ER stress, another ER transmembrane protein, ATF6, is transported to the Golgi where it is proteolytically cleaved to generate the N terminal portion of ATF6 to be released into the cytoplasm. The cytoplasmic portion of ATF6 contains a nuclear localization signal and a transcription activation domain. Thus, the cleaved ATF6 fragment then enters into the nucleus and acts as an active transcription factor for a number of UPR target genes [23,24].

Given the intracellular localization of CTE-associated mutant EpCAM and EpCAM’s role as a secretory pathway protein expressed on the cell surface in the normal intestine, interrogating the activation status of UPR signaling branches might provide a first step towards understanding CTE disease pathophysiology. We, therefore, hypothesized that the mutation of EpCAM occurring in CTE causes the aberrant accumulation of the protein in the ER and activates UPR. Hence, cellular localization of the mutant EpCAM and the three mechanistic arms of UPR were evaluated in our murine model of CTE.

## 2. Materials and Methods

### 2.1. Animals

All animal experiments were performed according to the instructions and guidelines of the University of California San Diego (UCSD) Institutional Animal Care and Use Committee (IACUC Protocol #S04100). Two previously described exon 4-deleted EPCAM mutant (EPCAM^Δ4/Δ4^) murine models, the neonatal murine model with constitutive expression of mutant EpCAM (Mutant) and the adult murine model with tamoxifen-inducible expression of mutant EpCAM (Ind. Mutant) were employed for the current study [9,25]. Although both models functionally represent CTE pathology, the two models differ from each other with respect to effects on the lifespan and generation of the mutation. The global constitutive neonatal mutant mice have limited postnatal lifespan (100% demise by postnatal day 6) whereas adult inducible mutant mice survive after induction of the mutation although they lose 10–20% of body weight over time [9,25]. As the name suggests, mice with the constitutive mutation have only mutant EPCAM expression. Adult inducible mice express mutant EpCAM only after induction of mutation with tamoxifen. For Ind. Mutant mice, mutant EpCAM was expressed upon oral gavage of tamoxifen as described earlier [25]. Littermates with wild type EPCAM (EPCAM^WT/WT^) served as controls (Control) for their respective neonatal mutant mice or adult Ind. Mutant mice (with tamoxifen treatment as appropriate). The constitutive mice and their respective controls were sacrificed from Day 1–3 postnatally. Both male and female mice were used in the study to avoid sex bias.

### 2.2. Cell Culture

To ensure our ability to detect a UPR response, T84 human colonic adenocarcinoma cells were utilized. Though colonic, rather than small intestinal, T84 cells were utilized as they are a well-studied model for polarized intestinal epithelial cells [26,27,28]. The cells were cultured in 1:1 Dulbecco’s modified Eagle’s medium/F12 Ham’s medium (Gibco, Waltham, MA, USA) with 15 mM l-glutamine (Gibco), 5% bovine calf serum (Sigma, St. Louis, MO, USA), 1% penicillin-streptomycin (Hyclone, Logan, UT, USA) and maintained as described earlier [25]. HEK293 cells were used as a control for XBP1 splicing, as described earlier [29]. T84 cells at 80–90% confluency were treated with 3 μM thapsigargin (Thap) (AdipoGen Life Sciences, San Diego, CA, USA) for 3 h as a positive control for the ER stress response [30]. Untreated T84 cells were used as negative control. After treatment, cells were collected for RNA and protein analyses. In order to confirm IRE1-mediated RIDD, IRE1 inhibitor 4μ8c (10 μM) [31] was administered along with 3 μM thapsigargin for 5 h.

### 2.3. Immunofluorescent (IF) Staining

Immunofluorescence studies were conducted with formaldehyde-fixed small intestinal tissue sections after deparaffination and antigen retrieval, as described previously [3]. Five percent goat serum was used as a blocking buffer. EpCAM and ATF6 antibodies were used at a dilution of 1:100, whereas GRP78/BiP antibody was used at a 1:200 dilution. Secondary antibodies were used at a 1:400 dilution in the blocking buffer. The slides were viewed via a Leica (Leica Microsystems, Buffalo Grove, IL, USA) confocal imaging system (DMI4000 B) using a 25× Plan-Apo 0.8 Numerical aperture with 63× objective lens, and the images were captured using LASX v4.1 Image Acquisition software. The co-localization of GRP78/BiP and EpCAM was analyzed using Pearson’s correlation coefficient through ImageJ (US National Institute of health, Bethesda, MD, USA) [32]. An average of 3 field correlation values were taken to calculate the co-localization index for each sample. Total ATF6 signal quantification was performed by measuring the fluorescence intensity of ATF6 in a region of approximately 10 epithelial cells in each of 4 distinct high-power fields from each murine and T84 sample. The areas of nuclei were then defined using Draq5. Quantitation of nuclear ATF6 was performed on 10 epithelial cells in each of 4 distinct high-power fields (40 nuclei per each control or mutant intestine). To quantitate the extent of ATF6 activation, the levels of nuclear and total ATF6 fluorescence were quantitated and the ratio was shown. Primary antibodies used for these experiments were: rabbit anti-EpCAM (E144) (ab32392, Abcam, Cambridge, UK), mouse anti GRP78/BiP (A10) (sc376768 Santa Cruz Biotechnology, Dallas, TX, USA), and rabbit anti-ATF6 (24169-1-AP, Proteintech, Rosemont, IL, USA). For the secondary antibodies, we used Alexa Fluor 488 goat anti-mouse (Thermo Fisher Scientific, Waltham, MA; for GRP78), and Alexa Fluor 568 goat anti-rabbit (Thermo Fisher Scientific for EpCAM and ATF6). Draq5 fluorescent probe (Thermo Fisher Scientific) was used to stain nuclei.

### 2.4. Electron Microscopy

Small intestinal tissue for electron microscopy was collected from both control and mutant neonatal mice. All the samples were prepared as described earlier using modified Karnovsky’s fixative [9] for at least 4 h and processed according to the protocol by the UC San Diego electron microscopy facility. Images were obtained at 10,000× magnification, focusing on the epithelial cells (enterocytes). In order to evaluate ER morphology and ultrastructure, at least 8 different fields per sample were taken for analysis. In each field, all of the measurable ER (at least 4 in any field) were included in the study. ER dimensions were quantified by measuring the area/perimeter of the ER using ImageJ.

### 2.5. Western Blotting

Total cell extracts of T84 cells and intestinal tissue were prepared by cell lysis using RIPA buffer (CST), followed by centrifugation (14,000× *g* for 15 min) at 4 °C, as described previously [25]. Total cell extracts were separated on a 12% SDS-PAGE, followed by transfer to a PVDF membrane (Immobilon-PSQ PVDF membrane, Millipore-Sigma; Burlington, MA, USA). Immunoblotting was performed as described earlier [3], using rabbit polyclonal antibodies to ATF4 (C-20; Santa Cruz Biotechnology 1:1000), phospho-eIF2α (P-eIF2α, Ser51, cat #9720), eIF2α (Cat #9720, both from CST), and ATF6 (Cat #24169-1-AP, Proteintech). Western blot signals blotted with mouse anti-β-Actin (A1978; Sigma-Aldrich, St. Louis, MO, USA; 1:5000) were used as a loading control. The band intensities from the Western blot images were analyzed with ImageJ after normalizing for the loading control and the fold change of protein expression in mutant models was calculated relative to the control group.

### 2.6. RT-PCR and Real-Time qRT-PCR

Total mRNA for reverse transcriptase (RT)-PCR and Real-Time quantitative (q)RT-PCR studies of both T84 cells and small intestine of control and mutant mice were isolated as per the manufacturer’s instructions using a quick RNA micro prep kit (Zymo research, Irvine, CA, USA) as described previously [25]. First-strand cDNA was synthesized from 300 ng of total RNA with iScript cDNA Synthesis kit (Bio-Rad) following the manufacturer’s protocol. The primers used for RT-PCR and Real-Time qRT-PCR in the current study are listed in Table 1. RT-PCR reactions were set up in the thermal cycler (Applied Biosystem) using Taq (AmpliTaq Gold, Applied Biosystems) polymerase with the following conditions (95 °C for 60 s, 58 °C for 30 s, 72 °C for 30 s) for 35 cycles followed by 5 min extension at 72 °C. The RT-PCR products were resolved in a 2.5% agarose gel. The percentage of spliced XBP1 mRNA was determined, as described earlier [29]. Real-time qRT-PCR reactions were set up using FastStart Universal SYBR Green Master Mix (Thermo Fisher Scientific) and thermal cycling was performed using a StepOnePlus (Applied Biosystems, Foster City, CA, USA) Real-Time PCR System using Step One software v2.0 (Applied Biosystems). All qRT-PCR reactions were performed in duplicate. The relative fold change of the respective gene was calculated after normalization to the housekeeping gene and comparison to the control group.

### 2.7. Statistical Analysis

Data are presented for at least eight biological replicates for the murine models and at least three independent experiments in case of UPR controls with T84 cells unless otherwise indicated. Error bars represent the standard deviation (SD) of the mean of the measured parameter. The statistical significance of differences between the two groups was calculated by Student’s unpaired *t*-test (Prism 7; GraphPad Software Inc., San Diego, CA, USA). Two-tailed *p* values of <0.05 were considered statistically significant. The *p* values were designated as ns (non-significant) *p* > 0.05; *, *p* < 0.05; **, *p* < 0.01, and ***, *p* < 0.001.

## 3. Results

### 3.1. Mutant EpCAM Accumulates in the ER

**Figure 1:**
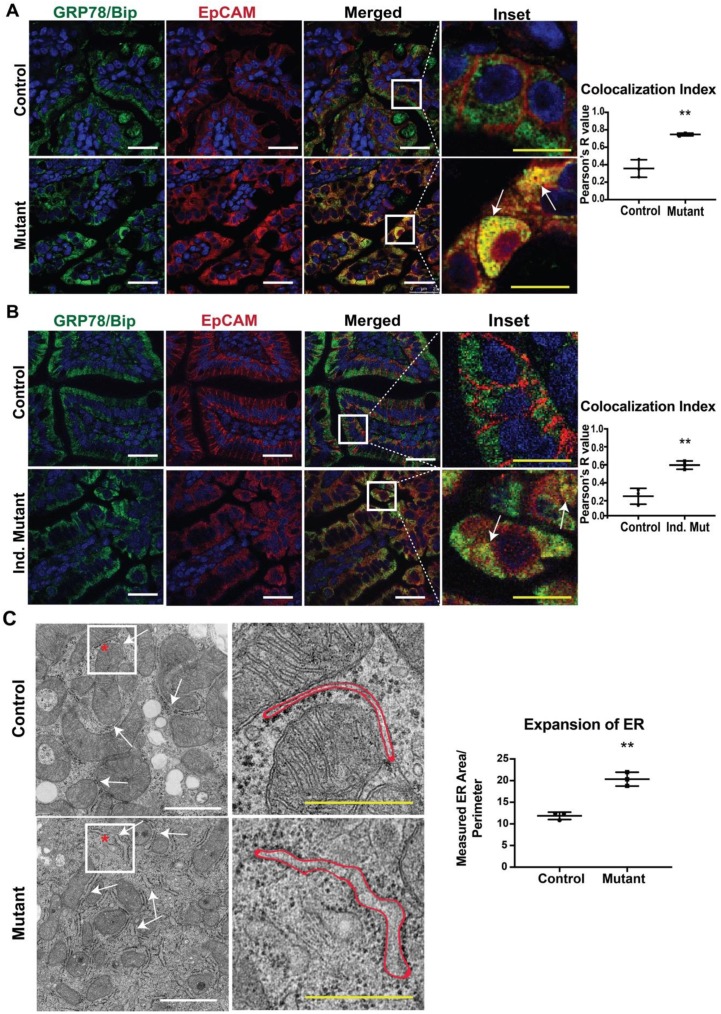
Co-localization of mutant epithelial cell adhesion molecule (EpCAM) with GRP78/BiP endoplasmic reticulum (ER) Marker: Representative confocal images of an intestinal tissue section from (**A**) neonates and (**B**) adult mice showing GRP78/BiP (green), EpCAM (red) and merged images of respective Control and Mutant/Inducible (Ind.) Mutant mice (*n* = 3). The right most panel depicts the enlarged inset image of the selected region from the merged images. Mutant EpCAM co-localizes with the major ER chaperone, GRP78/BiP, as depicted by yellow color (marked with arrows in inset). White scalebar represents 25 μm and the yellow scalebar represents 10 μm in all of the images for (**A**) and (**B**). The graphs on the right depict colocalization indices in terms of Pearson’s coefficient R from *n* = 3 samples. Each dot represents an average of 3 field values per sample. **, *p* < 0.01 from student *t*-test. (**C**) Representative electron microscopy images of neonatal control and mutant small intestinal tissue. The arrows in the left panel of images denote the ER and the right panel shows the higher magnified images of the inset. The red outline represents the area of an ER taken into consideration for ER expansion analysis. White scalebar represents 1 μm and the yellow scalebar represents 500 nm. The graph describes the average ER area/perimeter from *n* = 3 samples. **, *p* < 0.01 from student *t*-test.

In our previous study, we reported that mice expressing mutant EpCAM recapitulated the disease phenotype, including the pathology of the intestinal epithelium [9]. In order to provide a molecular understanding of the observed mutant phenotypes, we performed immunofluorescence (IF) studies in small intestinal tissue from both CTE murine models with antibodies specific for EpCAM and a major ER chaperone, GRP78/BiP (Figure 1A,B). No significant differences were observed in GRP78/BiP staining intensity or localization in either mutant model (neonatal, Figure 1A and adult, Figure 1B) when compared to the respective control. The intestines of control mice from both models showed significant EpCAM localization to the basolateral surface of the cells, consistent with the cell surface expression of EpCAM. GRP78/BiP (neonatal, Figure 1A and adult, Figure 1B) [33] was localized to the ER and did not co-localize with EpCAM. In contrast, mutant EpCAM was no longer expressed on the cell surface in the epithelial cells of neonatal mutant mice, consistent with our previous study (Figure 1A lower panel) [9]. Similarly, mutant EpCAM was also localized within the epithelial cells of adult mice and not expressed at the cell surface (Figure 1B lower panel). Importantly, staining for EpCAM was co-localized with that for GRP78/BiP (Figure 1A,B, merged, inset and co-localization index) in both neonatal and adult mutant intestines. The co-localization indices in both mutant models were found to be nearer to +1 (perfect co-localization) while the colocalization index of the controls were towards 0 (no co-localization). These results reveal that mutant EpCAM accumulates in the ER in both the neonatal constitutive mutant (Mutant) and adult inducible mutant (Ind. Mutant) models.

Our data showing the co-localization of mutant EPCAM with an ER chaperone (GRP78/BiP) led us to further study the ultrastructure of ER via electron microscopy (EM). To evaluate whether accumulated mutant EpCAM causes any changes to ER ultrastructure, murine small intestinal tissue sections were studied. A systematic review of the morphology of the rough ER revealed that ER membrane/tubules/sheets were significantly expanded in the mutant murine tissue sections compared to control tissue sections. Quantification of the area/perimeter of the ER confirmed that the ER is significantly dilated in mutant murine samples compared to control (Figure 1C). These data further strengthen our conclusion that mutant EpCAM resides in the ER, resulting in dilated/expanded ER tubules.

### 3.2. Activation of IRE1-Mediated RIDD Pathway

**Figure 2:**
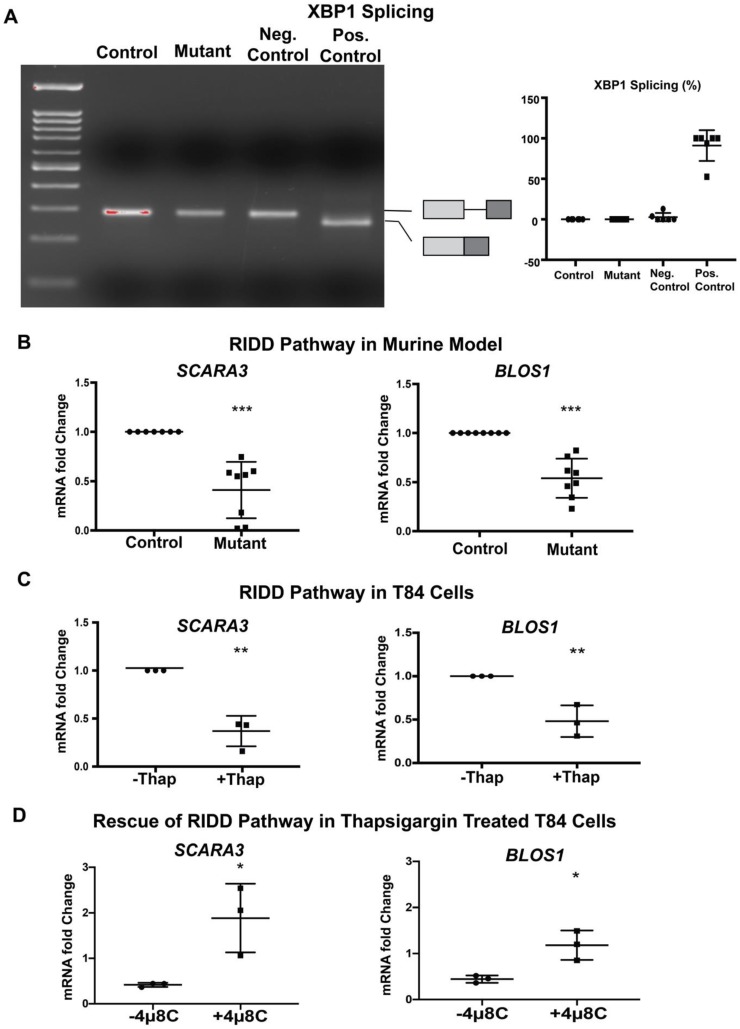
Inositol-requiring enzyme 1 (IRE1)-mediated pathway: (**A**) Representative gel image of XBP1 RT-PCR from Control and Mutant murine model as well as negative (Neg.) and positive (Pos.) control. The schematic of X-box binding protein 1 (XBP1) and spliced XBP1 is shown in the respective position of the gel image. The graph (right panel) shows the percentage of spliced XBP1 from *n* = 6 samples. XBP1 splicing was calculated by spliced XBP1/(spliced + unspliced XBP1 mRNA) × 100. (**B**) mRNA expression of the regulated IRE1-dependent decay (RIDD) pathway (*SCARA3*, *BLOS1*) in the intestinal tissue lysate of Control and Mutant murine model (*n* = 8). (**C**) mRNA expression of the RIDD pathway (*SCARA3*, *BLOS1*) in T84 cells with (+)/without (−) thapsigargin (Thap) treatment (*n* = 3). (**D**) mRNA expression of the RIDD pathway (*SCARA3*, *BLOS1*) in the presence (+) or absence (−) of IRE1α inhibitor 4μ8c. *, *p* < 0.05, **, *p* < 0.01, ***, *p* < 0.001 by student *t*-test in all of the results.

Our finding that mutant EpCAM accumulates in the ER (Figure 1) suggested the possibility that ER homeostasis was disrupted in murine CTE models. Further studies were performed in order to evaluate whether UPR was activated in response to mutant EpCAM accumulation. We first tested the activation of IRE1 signaling by examining the splicing of XBP1 mRNA [34,35]. Reverse transcription followed by PCR (RT-PCR) using primers that encompass the UPR intron was performed (Figure 2A) using intestinal tissue from mice. RNA isolated from both mutant and control mice demonstrated a PCR fragment size consistent with the unspliced form of XBP1, demonstrating that the IRE1 pathway was not significantly activated in mutant EpCAM intestinal cells. The size of this PCR product was identical to that from unstressed HEK293T cells. In contrast, treatment of HEK293 cells with a well-established ER stress inducer, thapsigargin, generated a PCR fragment smaller than that from unstressed cells, corresponding to spliced-XBP1 (sXBP1).

In addition to XBP1 splicing, activated IRE1 RNase has been reported to cleave a subset of mRNAs associated with the ER [20]. Using Real-Time qRT-PCR, we found that the steady-state levels of *SCARA3* and *BLOS1* mRNAs were significantly decreased in the mutant mice small intestine compared to control intestine (Figure 2B). Since both *SCARA3* and *BLOS1* are major targets of the RIDD pathway, our data suggest the active involvement of IRE1-mediated RIDD pathways in the tissue from mutant mice. The extent of the decrease in both RIDD substrate mRNA levels (*SCARA3* and *BLOS1*) was equivalent to that induced by thapsigargin in T84 cells (Figure 2C). These data indicate the partial involvement of IRE1 mediated UPR activation in a murine CTE model.

During investigation of the IRE1 pathways, our data show interesting results in mutant mice. While the IRE1 mediated RIDD pathway was activated, IRE1 mediated XBP1 splicing was not observed. This result led us to conduct further investigations with the IRE1 inhibitor, 4μ8c, in T84 cells to ensure degradation of *BLOS1* and *SCARA3* mRNA’s is medicated by IRE1. As expected, thapsigargin treated T84 cells showed significant downregulation in *BLOS1* and *SCARA3* mRNA when compared to the untreated group. However, when T84 cells were treated with thapsigargin together with 4μ8c, there was a rescue of *BLOS1* and *SCARA3* mRNA expression back to levels comparable to the untreated group (Figure 2D). These data further confirm the IRE1 specificity of RIDD activation.

### 3.3. PERK-Mediated Pathway Activation

**Figure 3:**
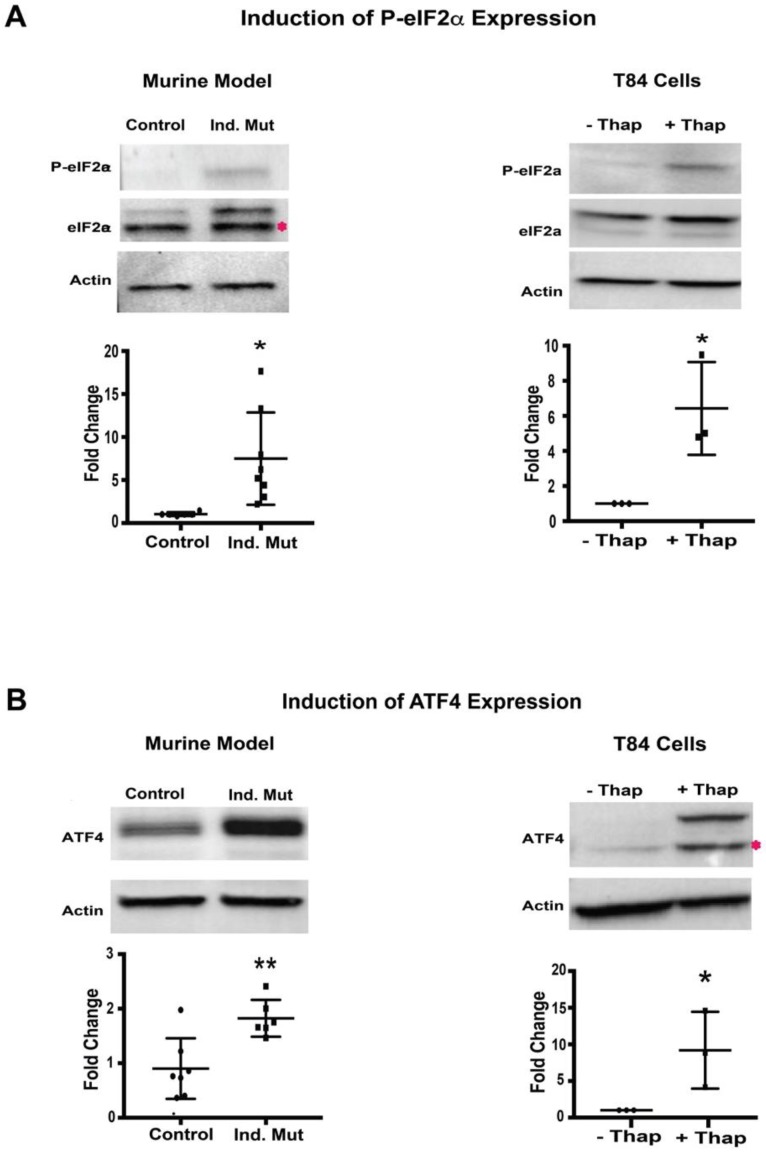
Protein kinase RNA-like endoplasmic reticulum kinase (PERK)-mediated pathway: (**A**) Western blot image and quantification of P-eIF2α, and eIF2α with respect to actin in Control/inducible (Ind.) Mutant adult murine intestinal tissue lysate (left panel) and T84 cells with (+)/without (−) thapsigargin (Thap) treatment (right panel). (**B**) Western blot images and quantification of ATF4 with respect to actin in adult murine intestinal tissue lysate (left panel) and T84 cells with (+)/without (−) thapsigargin (Thap) treatment (right panel). *, *p* < 0.05, **, *p* < 0.01 by student *t*-test.

Next, we evaluated the PERK signaling branch of UPR in a murine CTE model. The activation of this arm was assessed as the phosphorylation status of eIF2α. Small intestine isolated from Ind. Mutant mice had a significant increase (around 5-fold) in the level of phosphorylated-eIF2α (P-eIF2α) compared to control mice (Figure 3A, left panel), while the expression of total eIF2α was unchanged. As expected, when ER stress was induced in T84 cells, there was also a significant increase in P-eIF2α despite similar expression of total eIF2α normalized to β-actin (Figure 3A, right panel). The extent of the increase in P-eIF2α expression in the small intestine of the inducible mice was similar to that induced by thapsigargin in T84 cells, suggesting the initiation of the PERK UPR signaling branch in affected tissues.

Phosphorylation of eIF2α was further characterized by evaluating the expression of its downstream transcription factor, ATF4 [22]. The expression of ATF4 was significantly upregulated in intestinal tissue from Ind. Mutant mice tissue compared to control mice (Figure 3B, left panel). As anticipated, a significant induction of ATF4 expression was also seen in thapsigargin treated T84 cells. These results are consistent with the increased levels of P-eIF2α seen in both settings. The finding of both increased P-eIF2α and ATF4 expression suggests strongly that activation of the PERK-mediated pathway of UPR in this murine CTE model.

### 3.4. Increased Nuclear Presence of ATF6

**Figure 4:**
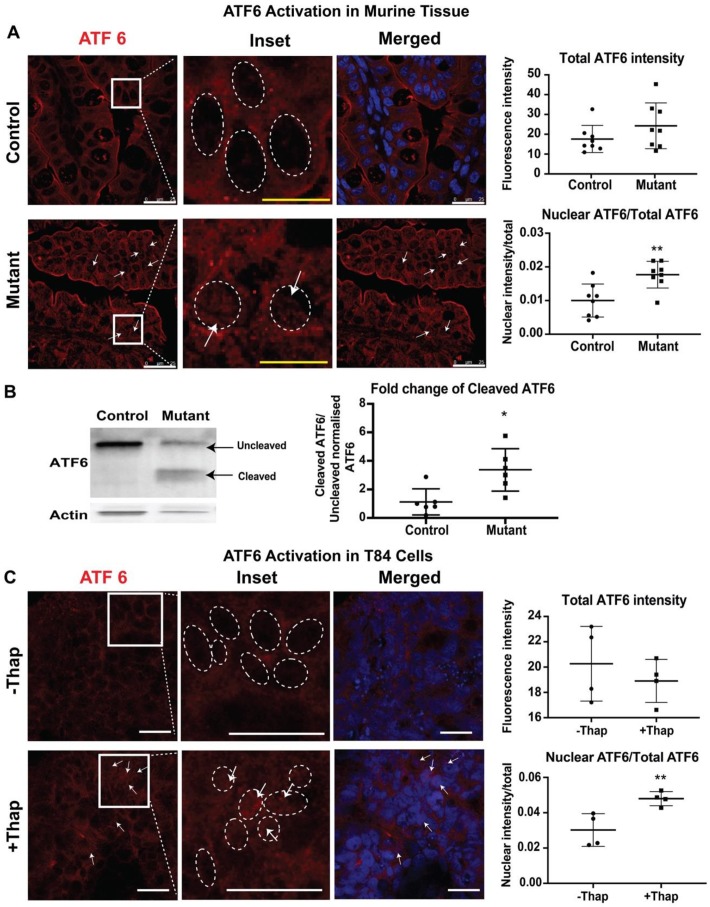
Activating transcription factor (ATF6) mediated pathway: Representative confocal images showing expression of ATF6 (red) and Draq5 staining for the nuclear DNA (blue) in (**A**) small intestinal sections of control and mutant mice. The middle panels show enlarged images of the cells within the sections marked by the white boxes. The dashed oval represents the nuclear border and arrows denote increased levels of the nuclear-localized ATF6. Upper graphs demonstrate total ATF6 fluorescence intensity. Lower graphs demonstrate nuclear ATF6 (*n* = 8) (**B**) Western blot image and quantification of uncleaved ATF6 (MW ~90 KDa) and cleaved ATF6 (MW ~50 KDa) with respect to actin in control/mutant murine intestinal tissue lysate (*n* = 6). Black arrow denotes the respective uncleaved and cleaved ATF6 fragment in the blot. (**C**) Representative confocal images showing expression of ATF6 (red) and Draq5 staining for the nuclear DNA (blue) in T84 cells with (+) or without (−) thapsigargin (Thap) treatment (*n* = 4). *, *p* < 0.05, **, *p* < 0.01 by student *t*-test in all the images. Scalebar represents 25 μm.

To study ATF6-mediated UPR signaling, the presence of cleaved ATF6 in the nuclei of intestinal tissues from neonatal mice was evaluated by immunofluorescence. In response to ER stress, the cytosolic domain of ATF6 migrates to the nucleus to induce a number of UPR target genes [24]. Nuclei were defined by Draq5 staining. In control tissues, ATF6 staining was seen within the cell, and nuclear ATF6 was detected, showing basal activation of ATF6. In contrast, a higher nuclear ATF6 signal intensity was observed in intestinal epithelial cells of mutant tissues compared to those in control tissues (Figure 4A). ATF6 activation was also tested in T84 cells. Treatment of T84 cells with thapsigargin caused increased levels of nuclear ATF6, while overall levels of ATF6 remained similar to those in unstressed cells (Figure 4B). Taken together, these results demonstrate activation of the ATF6-signaling pathway in CTE mice.

Nuclear ATF6 immunofluorescence suggests the migration of cleaved ATF6 into the nucleus, indicating the involvement of the ATF6 mediated pathway. In order to confirm the activation of ATF6 pathways in the CTE murine model, the active or cleaved fragment of ATF6 was further evaluated by western blot analysis. The 50 kDa cleaved fragment of ATF6 is generated when the ER is stressed and migrates to the nucleus. Whole intestinal tissue lysate of the mutant mice showed a significant increase in the expression of cleaved ATF6 when compared with control littermates. The staining and western blot data both confirm the active involvement of ATF6 to elicit UPR in the CTE model.

## 4. Discussion

As a step towards providing a better understanding of CTE, we previously developed mutant EpCAM murine models based on mutations found in a subset of CTE patients. These models pathologically and functionally recapitulate characteristics of human CTE [9,25]. Studies with these models and knowledge from other studies have enhanced the understanding of the CTE disease phenotype at cellular and molecular levels. Previous work suggests that EpCAM’s role in CTE may be multi-pronged, involving interactions with Claudin-7, SPINT2 and matriptase [9,36,37,38]. Both patients with CTE as well as murine models of the disease have varying phenotypes and severity of presentation, implying that deeper investigations of EpCAM’s role in the disease were needed. In the current study, we elucidated how the expression of mutant EpCAM itself may be involved in disease pathophysiology. Here, we provide evidence that mutant EpCAM, at least in our CTE murine models, accumulates in the ER and thus activates UPR, in order to alleviate ER stress.

The ER in mutant mice was found to be dilated, which is likely due to the accumulation of mutant EpCAM that could not leave the ER lumen, resulting in ER stress. The expansion of the ER has been reported in several ER stress conditions [39]. Thus, in line with the literature, our data are consistent with UPR activation due to the presence of mutant EpCAM in the murine CTE model. Our findings of co-localization of the ER lumen-resident chaperone GRP78/BiP with mutant EpCAM and dilated ER strongly argues the accumulation of mutant EpCAM in the ER. Protein modeling studies of the exon 4 deletion EpCAM mutant (EpCAM Δ4) predicts that the deletion of the core region of EpCAM contributes to the unfolding of the protein [6]. Accumulation of the mutant protein is likely to impose a significant burden on ER homeostasis. In this regard, many intestinal epithelial cell (IEC) lineages, such as Paneth cells, goblet cells, and enteroendocrine cells, are programmed to secrete high levels of antimicrobial proteins, mucins, and hormones, respectively. Folding of many of these secretory pathway proteins as well as post-translational modifications such as disulfide bonds and N-glycosylation, all have to occur within the ER. Thus, even a slight diminution of ER functions might be exacerbated in the intestinal epithelium where secretory demands are high, rendering the intestine especially sensitive to ER stress. Ultimately, in non-secretory cells, UPR activation might suffice to re-establish ER functional homeostasis. However, for specialized secretory cells, challenged with mutated secretory proteins, UPR activation alone may not meet such demands. Indeed, the vulnerability of secretory cells to ER stress may account for the decreased numbers of Paneth and goblet cells seen in CTE patients and the CTE murine model [40]. Thus, alterations in mucosal homeostasis in CTE and models of the disease are likely due, at least in part, to aberrant expression of mutant EpCAM.

Ultimate decisions about cell fate are thought to rest on the magnitude of ER functional recovery upon activation of three UPR signaling branches. Our data show that IRE1-mediated pathways are partially upregulated in EpCAM mutant tissues. Intestinal samples from mutant mice showed only unspliced XBP1 mRNA, and thus no activation of IRE1 dependent XBP1 mRNA splicing. In contrast, significant reductions in steady state levels of *BLOS1* and *SCARA3* mRNA suggest activation of IRE1-induced RIDD activity [41]. While the exact in vivo roles of RIDD remain elusive, RIDD activation has increasingly been reported in certain diseases such as Japanese encephalitis viral infection [42], underscoring the functional significance of RIDD. Activation of RIDD pathways not only alleviates ER stress by degrading ER-resident mRNAs, but also, along with activated XBP1, regulates cell fate [43,44]. Our data reveal, UPR due to mutant EpCAM has an interesting phenotype in that evidence of RIDD activation is detected, while XBP1 splicing is not observed in the CTE murine model. The involvement of the IRE1 mediated pathway in downregulating *BLOS1* and *SCARA3* mRNA (RIDD) pathway was confirmed with the inhibitor 4μ8c [31]. Typically, both outcomes are mediated through IRE1 endoribonuclease activity, although splicing of XBP1 and RIDD pathways are reportedly two independent outputs of IRE1-mediated UPR activation [45]. Although beyond the scope of this study, one may speculate differential activation of XBP1 splicing and RIDD might be attributable to signaling by the IRE1β isoform rather than IRE1α. The ER stress sensor IRE1β is uniquely expressed in mucosal epithelia, including in the intestinal epithelium [46]. Further, while both IRE1β and IRE1α possess endonuclease activity, some recent studies suggest IRE1β negatively regulates IRE1α signaling to attenuate ER stress-induced XBP1 splicing [47]. Thus, IRE1β in intestinal epithelial cells might suppress IRE1α-mediated XBP1 splicing in IECs of the CTE murine model.

Increased levels of phosphorylated eIF2α in epithelial cells from the inducible EpCAM mutant transgenic mice as well as ATF4 expression provide evidence that the kinase that phosphorylates eIF2α is activated in CTE. Phosphorylation of eIF2α leads to global attenuation of protein translation in order to release the protein load due to ER stress [48]. A significant increase in nuclear ATF6 levels was also observed in mutant cells, confirming the activation of the ATF6-mediated pathway in our CTE model. Recently, we have reported that ATF6 activation occurs via either its ER luminal domain or transmembrane domain, leading to two independent responses [49]. Further studies will be required to determine how ATF6 becomes activated in CTE.

Though all three branches of UPR are found to be at least partially or even fully activated in the murine CTE model, it was interesting to note the unchanged expression of the ER chaperone, GRP78/BiP, between control and mutant mice. We speculate several scenarios that may account for this result. The steady-state expression of GRP78/BiP might be sufficient to bind to mutant EpCAM and thus would not require upregulation in our mutant model. Another explanation could be the involvement of other stress-related ER chaperones, other than GRP78/BiP, in binding with mutant EpCAM. Future studies with different ER markers and stress chaperones could be performed in CTE to shed light on this finding.

Our findings suggest that CTE should be added to diseases such as amyotrophic lateral sclerosis (ALS) [50], retinitis pigmentosa (RP) [51], and neurodegenerative diseases [52] as a condition where the UPR plays an important pathophysiological role. Thus, investigation of UPR in CTE patient tissues will be valuable. Amongst gastrointestinal pathologies, ER stress has been reported in IBD. IBD and CTE both affect the gut mucosa. In contrast to IBD, where ER stress modulates the inflammatory response and thus affects the mucosal barrier, CTE involves an alteration in IEC homeostasis. The current study, therefore, links a childhood non-inflammatory intestinal mucosal disease with UPR activation using a murine model. ER stress may be an important mechanism in CTE that arises due to the mutation of EpCAM. The findings in this study further lay the foundation for studies that might, for example, assess the efficacy of UPR modulators in CTE models and eventually patients, who are a vulnerable population without any direct treatment options at this time. Additionally, future studies might correlate the extent of the UPR response with the severity of disease and associated patient morbidity and mortality. Our study provides initial findings implicating UPR as a pathogenic mechanism in CTE, identifying a targetable pathway and contributing to a much-needed expansion of this field.

## Figures and Tables

**Table 1:** List of Primers.

Gene	Forward Primer (5′–3′)	Reverse Primer (5′–3′)
BLOS1	AGCGTTGGTGGATCACCTC	CACCATTCCAATCCACTGGC
SCARA3 (Mouse)	TGCATGGATACTGACCCTGA	GCCGTGTTACCAGCTTCTTC
SCARA3 (Human)	CCGCTGCCAGAAGAACCTAT	TGTCTTCGGAGAGAGAGTCCA
XBP1	TTACGGGAGAAAACTCACGGC	GGGTCCAACTTGTCCAGAATGC

Table showing the lists and the nucleotide sequences of primers that were used in the current study.
